# Atypical Roles of the Chemokine Receptor ACKR3/CXCR7 in Platelet Pathophysiology

**DOI:** 10.3390/cells11020213

**Published:** 2022-01-09

**Authors:** Madhumita Chatterjee

**Affiliations:** Department of Cardiology and Angiology, University Hospital Tübingen, 72076 Tübingen, Germany; madhumita.chatterjee@med.uni-tuebingen.de; Tel.: +49-7071-29-82888

**Keywords:** platelet, ACKR3/CXCR7, thrombosis, thrombo-inflammation, anti-platelet therapy, cardiovascular disease, coronary artery disease

## Abstract

The manifold actions of the pro-inflammatory and regenerative chemokine CXCL12/SDF-1α are executed through the canonical **GP**rotein**C**oupled**R**eceptor CXCR4, and the non-canonical ACKR3/CXCR7. Platelets express CXCR4, ACKR3/CXCR7, and are a vital source of CXCL12/SDF-1α themselves. In recent years, a regulatory impact of the CXCL12-CXCR4-CXCR7 axis on platelet biogenesis, i.e., megakaryopoiesis, thrombotic and thrombo-inflammatory actions have been revealed through experimental and clinical studies. Platelet surface expression of ACKR3/CXCR7 is significantly enhanced following myocardial infarction (MI) in acute coronary syndrome (ACS) patients, and is also associated with improved functional recovery and prognosis. The therapeutic implications of ACKR3/CXCR7 in myocardial regeneration and improved recovery following an ischemic episode, are well documented. Cardiomyocytes, cardiac-fibroblasts, endothelial lining of the blood vessels perfusing the heart, besides infiltrating platelets and monocytes, all express ACKR3/CXCR7. This review recapitulates ligand induced differential trafficking of platelet CXCR4-ACKR3/CXCR7 affecting their surface availability, and in regulating thrombo-inflammatory platelet functions and survival through CXCR4 or ACKR3/CXCR7. It emphasizes the pro-thrombotic influence of CXCL12/SDF-1α exerted through CXCR4, as opposed to the anti-thrombotic impact of ACKR3/CXCR7. Offering an innovative translational perspective, this review also discusses the advantages and challenges of utilizing ACKR3/CXCR7 as a potential anti-thrombotic strategy in platelet-associated cardiovascular disorders, particularly in coronary artery disease (CAD) patients post-MI.

## 1. Introduction

Platelets are versatile cells in circulation that relentlessly patrol the blood vessels as sentinels detecting vascular injury, mount hemostasis, sense pathogenic intrusion and execute thrombo-inflammatory response in coordination with the immune system [1,2,3,4]. The role of platelets in instigating thrombotic occlusion of the coronary artery causing thrombo-ischemic events like MI, infiltration of activated platelets into the affected myocardium leading to either deleterious or regenerative consequences are widely investigated [5,6,7]. The balance between these opposing actions and the resultant outcome depends on the contribution of activated platelets and platelet-derived factors. Platelets escalate an acute inflammatory response following MI in compliance with monocytes and neutrophils; and subsequently prompt resolution of the inflammatory process leading to myocardial regeneration and functional recovery [7]. Platelets are a readily available cargo of functionally opposing inflammatory (CXCL4, CCL5) and anti-inflammatory (IL-10) cytokines/chemokines, angiogenic (VEGF) as opposed to anti-angiogenic (endostatin) mediators, growth factors (PDGF, TGF-β) [8], which can be delivered and enriched locally at the site of myocardial damage as they infiltrate. Thereby platelets may direct the course of cardiac inflammation, regeneration or fibrosis [7,9,10]. Platelets are an enriched source of TGF-β, which is a major pro-fibrotic factor that triggers collagen synthesis and mediates tissue remodeling [11]. At the same time activated platelet-derived CXCL12/SDF1α has distinctive regenerative potential in facilitating the migration of endothelial progenitor cells [12,13,14], and in promoting the differentiation of monocytes into reparative M2-macrophages instead of a pro-inflammatory M1-phenotype [15,16]. Platelet surface expression of CXCL12/SDF1α is significantly enhanced in ACS patients, particularly in those with reduced (<55%) left ventricular ejection fraction (LVEF) [17]. Platelet surface-associated CXCL12/SDF1α correlates positively with the extent of platelet activation (e.g., CD62P) signifying granular release; it also correlates with the number of circulating CD34^+^ progenitor cells and CD34^+^CD133^+^ endothelial progenitor cells (EPCs) [17]. Platelets adherent on endothelial cells express substantial amounts of CXCL12/SDF1α, which effectively recruits CD34^+^ progenitor cells, while activated platelet derived CXCL12/SDF1α promotes subsequent differentiation of human CD34^+^ progenitor cells into EPCs [13]. Percentage of circulatory platelet-progenitor cell aggregates is significantly enhanced in ACS, particularly in patients with ST-elevation myocardial infarction (STEMI), who also exhibit a CXCL12/SDF1α enriched platelet surface. Following acute myocardial infarction (AMI), patients with increased platelet-CD34^+^-aggregates assessed at baseline show less deteriorated infarct size and better LVEF-recovery within a 3-month follow-up period. Aggregate formation with platelets facilitates the adhesion of CD34^+^ progenitors onto extracellular matrix and endothelial cells. Therefore, platelet-progenitor cell aggregates are suggested in supporting domiciliation of CD34^+^ progenitor cells to the affected microvasculature after AMI [18,19]. Platelet surface-associated or activated platelet derived CXCL12/SDF1α acts as a prominent mediator in influencing these regenerative processes [19,20]. 

CXCL12/SDF1α either from autocrine or paracrine cellular sources also exerts a significant influence on human and murine platelet functions and survival by engaging its receptors CXCR4 and ACKR3/CXCR7 [8,15,21]. Over the years we have explored the pathophysiological relevance of platelet CXCL12-CXCR4-CXCR7 axis in the context of CAD. Like CXCL12/SDF1α, [22] platelets from CAD patients exhibit enhanced surface expression of CXCR4 and ACKR3/CXCR7 [23] as compared to healthy subjects. ACKR3/CXCR7 surface expression is particularly increased in ACS as compared to stable-CAD patients. It also correlates with platelet surface-associated CXCL12/SDF1α [23]. Moreover, platelet ACKR3/CXCR7 surface expression ascertained upon admission immediately following an acute thrombo-ischemic event, is associated with significantly improved functional recovery (LVEF) and prognosis [23,24], evaluated during a hospital stay of 5 days, and over a 6 month follow-up period. The prognostic significance of platelet CXCR4 surface expression at baseline, which though comparable between stable CAD and ACS patients becomes more prominent after a 1-year follow up. Symptomatic CAD patients exhibiting lower levels of platelet CXCR4 surface expression at baseline succumb to all-cause death and/or MI to a significant extent. Within the same time-frame, although lower surface expression of ACKR3/CXCR7 is found to be associated with all-cause mortality in symptomatic CAD patients, its prognostic association with death on account of recurrent MI appears to be less pronounced [25]. Evidence on platelet CXCR4 and ACKR3/CXCR7 surface expression from a longitudinal cohort is currently lacking and could be investigated to ascertain their impact on long term prognosis. At present, one can only speculate a few possibilities. Expression of ACKR3/CXCR7 is significantly increased at the infarct border zone of myocardium [26,27,28], and peri-infarct regions of the brain [29] in animal models [27,28,30,31] and humans alike [26,28,32]. Firstly, target organ (e.g., myocardium) specific expression of ACKR3/CXCR7 might either override or be complemented by the impact of platelet ACKR3/CXCR7 in the repair and regenerative mechanisms. Since activated platelets infiltrate ischemia affected myocardium [5,7,33,34], they can actively regulate the balance between inflammation vs regeneration [7]. The extent to which platelet ACKR3/CXCR7 may influence this functional outcome, superseding the acclaimed regenerative drive of ACKR3/CXCR7 highly expressed in cardiomyocytes [26] and endothelial cells [27], is uncertain. Cardiomyocytes, like circulating platelets [22], exhibit increased expression of CXCL12/SDF1α following MI, which significantly influences the migration of CXCR4 and ACKR3/CXCR7 expressing cardiac stem cells with regenerative potential [35] as observed in murine models. Secondly, it has been shown in CAD patients that ACKR3/CXCR7 mediated signaling involving Erk regulates the re-endothelialization capacity of regenerative endothelial outgrowth cells (EOC), which play an important role in revascularization of the ischemic organ, quite independent of any influence from circulating platelets. However, EOC from CAD patients show reduced expression of ACKR3/CXCR7 as compared to healthy subjects [36]. The differential impact of platelet CXCR4 and ACKR3/CXCR7 on long-term (1-year) prognosis for recurrent MI [25] could be due to the difference in their expression pattern on platelets. CXCR4 surface expression is relatively more prominent under physiological conditions [37], while ACKR3/CXCR7 may be transiently induced post-MI [23] by circulatory or platelet-associated CXCL12/SDF1α in ACS patients [22,38] through receptor trafficking, as discussed next. 

## 2. The Dichotomy of CXCR4 and ACKR3/CXCR7 in Platelets

### 2.1. The Typical and the Atypical: Differential Trafficking of CXCR4-CXCR7 in Platelets 

The relative surface abundance of Gαi-coupled canonical CXCR4 and non-canonical ACKR3/CXCR7 on platelets and immune cells (e.g., monocytes [16] and lymphocytes [39]) can directly influence their extent of functional engagement in different pathophysiological processes. CXCR4 and ACKR3/CXCR7 are constitutively expressed at transcript and protein levels in human platelets, but CXCR4 surface expression predominates over ACKR3/CXCR7 [37,40] as evident from the relative difference in the percentage of CXCR4^+^ (over 90%) and ACKR3/CXCR7^+^ (around 50%) human and murine platelets [37]. The copy number for CXCR4 on human platelets is calculated to be around 2600, which suggests that it is fairly expressed on platelets [41]. The relative copy number of ACKR3/CXCR7 on platelets under a basal state or in the presence of ligands is currently warranted. We have demonstrated that CXCR4 and ACKR3/CXCR7 are shared by pro-inflammatory physiological ligands- CXCL12/SDF-1α and microphage migration inhibitory factor (MIF), while CXCL11 is an exclusive ligand for ACKR3/CXCR7 [37,40]. Presence of CXCL12/SDF-1α, CXCL11 and MIF in the immediate microenvironment of platelets may induce a dynamic alteration in CXCR4 and ACKR3/CXCR7 surface expression as CXCR4 is internalized and ACKR3/CXCR7 is preferentially translocated to the platelet surface in response to CXCL12/SDF-1α [37] but not CXCL11 or MIF [40]. 

CXCL12/SDF-1α-induced CXCR4 internalization precedes in effect and is a pre-requisite for ACKR3/CXCR7 surface externalization. Therefore, it is counteracted by a blocking antibody directed against CXCR4 or by CXCR4-antagonist AMD3100. Internalization of CXCR4 in human platelets in vitro occurs as early as 5 min, while externalization of ACKR3/CXCR7 evidently requires more time and is significantly in effect from 30 min. Administration of CXCL12/SDF-1α drives internalization of CXCR4 within an hour while externalization of ACKR3/CXCR7 on murine platelets requires 2 h [37]. This bidirectional shuttling of CXCR4 and ACKR3/CXCR7 is executed through Erk, which interacts with and phosphorylates the intracellular molecular chaperone Cyclophilin A (CyPA). Both CXCR4 and ACKR3/CXCR7 associated with CyPA as seen in co-immunoprecipitation studies performed with human platelets. The peptidyl prolyl transferase (PPIase) activity of CyPA is essential for ACKR3/CXCR7 translocation to the platelet surface; therefore, it is counteracted by a CyPA inhibitor NIM-811 in human platelets, and ablated in platelets from *Cypa*^−/−^ mice. Ubiquitination of ACKR3/CXCR7 determines its shuttling between the cytosol and the cell membrane [42]. CXCL12/SDF-1α induces ubiquitination of ACKR3/CXCR7 involving E1-ligase activity, and translocates ACKR3/CXCR7 to the platelet surface [37], an effect validated in both human and murine platelets. MIF induces CXCR4 internalization like CXCL12/SDF-1α, but cannot transduce intracellular signals (i.e., downstream Erk phosphorylation) required for ACKR3/CXCR7 externalization in human platelets. This is because platelets lack CD74, which essentially acts as a co-receptor for MIF along with CXCR4 [40,43]. The ACKR3/CXCR7-specific ligand CXCL11 does not affect CXCL12/SDF1α-induced CXCR4 internalization, but internalizes ACKR3/CXCR7 in human platelets and may counteract CXCL12/SDF1α-induced ACKR3/CXCR7 externalization, if present simultaneously in the microenvironment [40]. Such an interplay of physiological ligands at the site of vascular inflammation either from another paracrine source or activated platelets themselves, may directly regulate surface availability of CXCR4 and ACKR3/CXCR7, and their functional engagement. Circulatory levels of CXCL12/SDF1α [38,44] and MIF [45] are elevated in ACS patients and are furthermore associated with progressive disease severity. Therefore, CXCL12/SDF1α and MIF in circulation may influence the relative surface expression and involvement of CXCR4-ACKR3/CXCR7 in modulating platelet response and survival potential [24]. Although both CXCR4 and ACKR3/CXCR7 serve as cognate receptors for CXCL12/SDF-1α and MIF, they are involved in quite contrasting functional responses, as discussed next [21].

### 2.2. The Pro-Thrombotic Attributes of Canonical CXCR4

**Figure 1:**
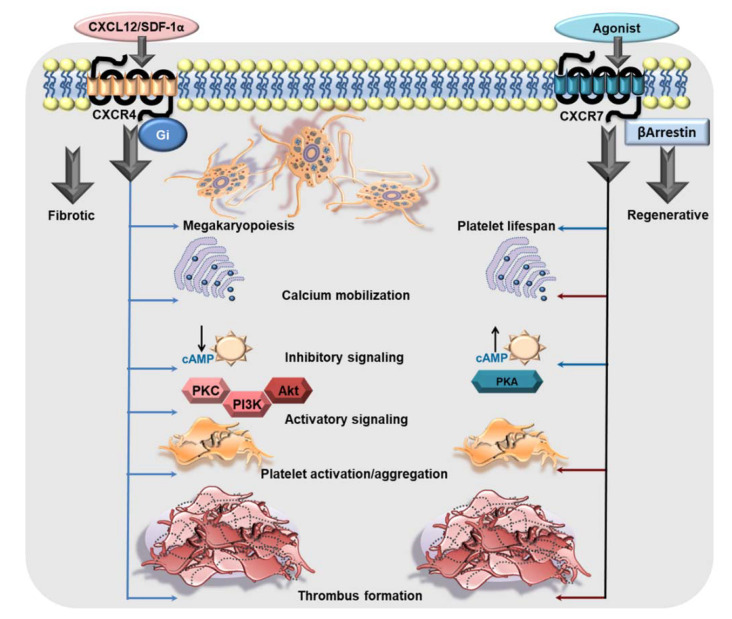
The functional dichotomy of CXCR4 and ACKR3/CXCR7 in platelets. CXCR4 is a Gαi-coupled GPCR which may exert its influence on platelet biogenesis (megakaryopoiesis), and pro-thrombotic response. CXCL12/SDF1α engages CXCR4 on platelets to trigger intraplatelet-calcium mobilization, the platelet activatory signaling pathway involving PI3K, Akt, which promotes platelet activation, aggregation, and a pro-thrombotic response. Moreover, Gαi-coupled CXCR4 induces a canonical signaling cascade following ligation by CXCL12/SDF-1α that imposes an inhibitory effect on AC and impedes the generation of cAMP. On the contrary, ACKR3/CXCR7 is an atypical GPCR, which does not engage a G protein but mostly β-arrestin. Physiological and pharmacological ligands of ACKR3/CXCR7 promote platelet survival. ACKR3/CXCR7-ligation by a pharmacological agonist (VUF11207) counteracts calcium mobilization induced by platelet-activating stimuli. ACKR3/CXCR7-ligation triggers the platelet inhibitory signaling cascade involving AC-cAMP-PKA, while counteracting activatory signaling mediators to impose an inhibition on platelet activation, degranulation, aggregation and pro-thrombotic response (red arrows denote an inhibitory effect on the process). This exemplifies the functional dichotomy of platelet CXCR4 and ACKR3/CXCR7 in mediating pro- and anti-thrombotic effects respectively.

CXCL12/SDF-1α, acting through CXCR4, can substantiate cell migration, proliferation, differentiation, and survival [15,16,46,47,48,49]. The CXCL12/SDF-1α gradient modulated by CXCR4 in the bone marrow vascular niche influences maturation of megakaryocytes, the formation of proplatelet extension and the release of platelets into the perfusing vessels, during platelet biogenesis, i.e., megakaryopoiesis (Figure 1) [41,50,51,52,53,54]. The pronounced effect of CXCL12/SDF-1α-CXCR4 axis on megakaryopoiesis, especially migration of immature megakaryocytes, has been simultaneously demonstrated by several groups in culture derived megakaryocytes from CD34^+^ bone marrow and cord blood-derived human progenitor cells [41,50,51,52,53]. However, mature megakaryocytes and peripheral blood platelets are also positive for CXCR4 transcripts and protein. Subsequent investigation from Rafii’s group demonstrated transmigration of CD34^+^-derived mature polyploid megakaryocytes through a monolayer of bone marrow endothelial cells across a CXCL12/SDF-1α and their ability to generate functional platelets [53]. Only recently has the contribution of CXCL12/SDF-1α enriched bone marrow reticular cells been elegantly elaborated in megakryopoiesis and proplatelet production in the mouse bone marrow sinusoidal microenvironment [54]. Much like their parental cells, mature human platelets can transmigrate through the endothelium following a CXCL12/SDF-1α gradient sensed through CXCR4, which is inhibited by CXCR4 antagonist AMD3100, Gαi inhibitor pertussis toxin, PI3K inhibitors LY294002 or wortmannin, and by disrupting actin polymerization with cytochalasin B [55,56]. Therefore, CXCL12/SDF-1α may actively sequester platelets by engaging platelet CXCR4 at the site of the vascular lesion, atheroprogression or at CXCL12/SDF-1α-enriched atherosclerotic plaques [57].

Experimental studies have shown that recombinant CXCL12/SDF-1α, mimicking that from a paracrine source, or from activated human platelets induces intracellular calcium mobilization, augments aggregation [57,58,59,60,61] and thrombotic potential exclusively through Gαi coupled CXCR4 (Figure 1) and not ACKR3/CXCR7 engagement. This response is counteracted by a CXCR4 blocking antibody, CXCR4-antagonist AMD3100 and pertussis toxin [57,58,59,61]. Interestingly, decreased platelet surface expression of CXCR4 results in impaired CXCL12/SDF1α triggered aggregatory response in patients with inherited thrombocytopenia [62]. CXCL12/SDF-1α can enhance adhesion of human platelets to extracellular matrix components like collagen and fibrinogen under static or arterial flow conditions [58], and increase platelet spreading in the presence of plasma lipoproteins (low density lipoprotein-LDL, oxidized low density lipoprotein-oxLDL) [63]. Besides, CXCL12/SDF-1α induced morphological changes leading to blebbing in platelets may also enhance blood coagulation [58]. In suspension, CXCL12/SDF-1α synergizes with collagen and convulxin triggered release of thrombo-inflammatory mediators like platelet derived growth factor (PDGF), sCD40L, by driving p38MAPK activation, and instigates release of phosphorylated-heat shock protein 27 (HSP27) from activated human platelets. CXCL12/SDF-1α is considered to be a weak platelet agonist and requires synergistic stimuli from the microenvironment in form of collagen, plasma components like serotonin (5-hydroxy tryptophan-5HT), thromboxane (TxA_2_), adenosine -di-phosphate (ADP), lipoproteins (LDL, oxLDL), or shear stress [58]. Therefore, as a pro-thrombotic mediator, CXCL12/SDF-1α is effective in platelet rich plasma (PRP) preparations, not washed human platelets [41,61], and under stirring but not non-stirring conditions [58,61]. CXCL12/SDF-1α can exert a synergistic effect with platelet activating stimuli to prompt phospholipase C activation, intracellular calcium mobilization, P-selectin exposure, α_IIb_β_3_-integrin activation, granular release from α- (e.g., CXCL4) and dense granules (e.g., ADP, ATP), generation of TxA_2_, all of which substantiate a pro-thrombotic response. At lower concentrations, CXCL12/SDF-1α may induce the primary phase of aggregation but instigates a full biphasic aggregation response with increasing concentrations [60]. CXCL12/SDF-1α synergistically enhances aggregation induced by subthreshold concentrations of strong agonists like thrombin and ADP, or relatively weaker agonists like 5HT, under arterial and lower shear stress conditions [58,60]. These effects are sensitive to CXCR4 and P_2_Y_12_ antagonism, apyrase, pharmacological inhibition of prostanoid production through COX-1 (aspirin), PI3K (wortmannin and LY29004), and tyrosine kinases (genistin) [57]. CXCR4 ligation by CXCL12/SDF1α triggers the platelet activatory signaling cascade involving PI3K, Akt, PDK1, GSK3β, causing myosin light chain phosphorylation [64]. Ex vivo supplementation of human blood with recombinant CXCL12/SDF1α, or its in vivo administration in mice enhances thrombus formation [59,63] over collagen-coated surface and in the injured artery, respectively [63] (Figure 1). CXCL12/SDF-1α also elicits a synergistic influence on the activating stimuli from plasma lipoproteins (LDL, oxLDL) to augment degranulation (CD62P surface expression), α_IIb_β_3_-integrin activation (PAC-1 binding), spreading, and thrombus formation [63]. This is of particular significance, as both platelet surface-associated CXCL12/SDF1α [12,17] and oxLDL [65], also intraplatelet levels of oxLDL are enhanced in ACS patients [63], and could thus account for platelet hyper-reactivity.

Acting through the Gαi-coupled canonical CXCR4, CXCL12/SDF-1α downregulates the production of platelet inhibitory cAMP and even antagonizes PGI_2_ analog-induced cAMP levels in human platelets [59]. Such actions may lower the activation threshold of circulating platelets under conditions where levels of circulatory CXCL12/SDF-1α are enhanced (e.g., in CAD patients) [38]. Moreover, activated platelet-derived CXCL12/SDF-1α acting in an autocrine manner, exclusively through CXCR4 and not ACKR3/CXCR7, promotes platelet aggregation, ATP release, intracellular calcium mobilization, thromboxane production and thrombus formation [59] (Figure 1). Such actions may foster a thrombotic response in a pro-inflammatory microenvironment where platelets accumulate. Therefore, antagonizing the CXCL12/SDF-1α-CXCR4 axis may offer therapeutic benefits [66,67,68] in regulating the pro-thrombotic drive of platelets. 

### 2.3. ACKR3/CXCR7 Boosts Platelet Survival 

ACKR3/CXCR7 is a prominent prosurvival receptor for primary and tumor cells alike. Aged and activated platelets are removed from circulation and eliminated in the reticulo-endothelial system and spleen [69], while precursor megakaryocytes release newly formed platelets into the bone marrow vasculature to maintain a steady turnover of functionally adept platelets in circulation. Since both aged and apoptotic platelets have reduced functional capacities [70], platelet response can not only be influenced by their activation status but by survival potential as well [71]. CXCL11, CXCL12 and MIF as physiological ligands of ACKR3/CXCR7 counteract apoptosis induced upon platelet activation by stimulatory agonists (e.g., thrombin), and that induced by pharmacological BH3-mimetic (ABT-737). Moreover, CXCL12/SDF-1α and MIF prolong the survival of circulating platelets through ACKR3/CXCR7 engagement [37,40] (Figure 1). Pharmacological inhibition of the Erk1/2 pathway (U0126), or CyPA-PPIase activity (NIM811) uncouples CXCR4-ACKR3/CXCR7 trafficking from the resultant anti-apoptotic effects of recombinant CXCL12/SDF-1α mediated through ACKR3/CXCR7 in human platelets in vitro. Administration of CXCL12/SDF-1α exerts a protective effect during subsequent activation-induced apoptosis ex vivo in murine platelets from wild type but not CyPA deficient mice. Anti-apoptotic effects of recombinant CXCL11 and MIF upon ACKR3/CXCR7 ligation are executed through downstream activation of the PI3K-Akt-pathway, which culminates in Akt-mediated phosphorylation and thereby inactivation of the pro-apoptotic effector protein BAD in human platelets. Therefore, a similar anti-apoptotic impact is abolished in murine platelets lacking Akt. These experimental evidences suggested the possibility that elevated plasma levels of CXCL12/SDF-1α [38] and MIF [45] in ACS patients might exert a similar influence on circulatory platelet survival. Subsequent clinical evidence from STEMI patients have indeed shown a significantly positive correlation between enhanced CXCR4, ACKR3/CXCR7 surface expression and survival potential of platelets. However, the relative functional efficacy of these aged platelets [70] in circulation as compared to newly released platelets through ongoing megakaryopoiesis [72], and also their regenerative involvement in tissue repair or functional recovery [73], remains to be ascertained.

### 2.4. Atypical Influence of ACKR3/CXCR7 on Thrombotic and Thrombo-Inflammatory Platelet Response

#### 2.4.1. Physiological CXCR7-Agonist MIF

Recombinant MIF does not exert any effect on the activation status of platelets either alone or synergistically in combination with activating stimuli, as ascertained by P-selectin (CD62P) surface exposure or degranulation of the proinflammatory chemokine CCL4 from α–granules [74,75] of human platelets. MIF does not regulate spreading of platelets over fibrinogen, which also rules out the possibility of an influence on α_IIb_β_3_-integrin activation. Therefore, unlike CXCL12/SDF-1α, MIF does not alter the aggregation response to ADP, or TxA_2_ analog in human platelets. Contrary to CXCL12/SDF-1α, MIF does not trigger intraplatelet calcium mobilization in TxA_2_ receptors agonist (U46619) treated platelets. However, both CXCL12/SDF-1α and MIF can desensitize platelets to increases in calcium transients triggered by ADP [74]. As a consequence of the prosurvival effect mediated through ACKR3/CXCR7, MIF attenuates exposure of pro-thrombotic phosphatidylserine on the surface of pro-coagulant platelets. Therefore, MIF exercises an anti-thrombotic effect in ex vivo flow chamber assay with human blood and retards arterial thrombus build-up in vivo in murine model. This inhibitory effect on thrombus stability is counteracted by blocking ACKR3/CXCR7 [40]. Although experimental evidences documented so far relate the functional dichotomy of CXCR4 as a pro-thrombotic and ACKR3/CXCR7 as an anti-thrombotic mediator (Figure 1), the contribution of ACKR3/CXCR7 to platelet pathophysiology warrants further in-depth investigation.

#### 2.4.2. Anti-Thrombotic Effects of Pharmacological Agonist VUF11207

**Figure 2:**
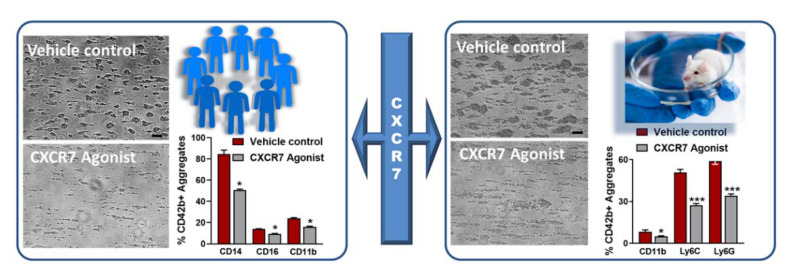
CXCR7-agonist reduces thrombotic response and thrombo-inflammatory platelet interaction with leukocytes ex vivo. Blood from healthy human subjects or mice pre-treated with vehicle control (1% DMSO) or CXCR7-agonist (100 µg/mL) for 30 min at room temperature is perfused through a transparent flow chamber (slit depth 50 μm) over a collagen (100 μg/mL)-coated surface at 1700 s^−1^ (human) or 1000 s^−1^ (murine) wall shear rate. After blood perfusion, the chamber is rinsed with PBS and images are captured from randomly chosen microscopic areas (Axiovert 200, Carl Zeiss, optical objective 20×). Phase contrast images for relative thrombus coverage shows decreased thrombus formation in CXCR7-agonist-treated human and murine blood. Bar = 20 µm. Effluent blood from the flow chamber is collected and analyzed for platelet-leukocyte aggregate formation by flow cytometry using platelet (CD42b) and leukocyte population specific surface markers (CD14, CD16, Ly6G, Ly6C). Bar diagram shows platelet-leukocyte aggregate formation is significantly reduced in the presence of CXCR7-agonist as compared to vehicle control. Data are mean ± S.E.M from 5 independent experiments performed with blood from n = 5 healthy subjects and n = 5 C57BL/6J mice. * *p* < 0.05, *** *p* < 0.001 with Mann–Whitney U-test.

Our previous investigation showed an enhanced surface availability of ACKR3/CXCR7 on circulating platelets in CAD patients [23,25], and the possibility of regulating thrombotic response through ACKR3/CXCR7, with MIF [40]. Therefore, we are currently corroborating the therapeutic implications of platelet ACKR3/CXCR7 as a potential anti-thrombotic drug target using a pharmacological CXCR7-agonist. Our recent finding shows that unlike MIF, pharmacological CXCR7-agonist (VUF11207) can interfere with platelet degranulation from both α-(CD62P surface expression, release of thrombo-inflammatory cytokines and chemokines) and δ-(CD63, ATP) granules, modulate α_IIb_β_3_-integrin activation, platelet interaction with and adhesion to physiological matrices (collagen and fibrinogen), and aggregation response to different stimuli [76] in both human and murine systems. Like MIF, CXCR7-agonist VUF11027 inhibits thrombus formation over collagen in ex vivo flow chamber assay and is efficacious in both human and murine blood (Figure 2). This offers the possibility of pre-clinical validation in murine models of arterial thrombosis, and in regulating thrombotic, thrombo-inflammatory platelet response following myocardial infarction/reperfusion injury (MI/RI) [76]. CXCR7-agonist administration reduces platelet degranulation, α_IIb_β_3_-integrin activation and thrombotic propensity 24 h post-MI; it also counteracts thrombus build-up, prolonging time to vessel occlusion following FeCl_3_-inflicted carotid artery injury. Ex vivo treatment of blood from ACS patients with CXCR7-agonist reduces degranulation, integrin activation, ADP, TRAP, collagen induced platelet aggregation and thrombus formation. This suggests the possibility of exploiting enhanced availability of ACKR3/CXCR7 on the platelet surface [23,25] as a potential anti-thrombotic drug target post-MI. Similar to the physiological ACKR3/CXCR7 ligand MIF, pharmacological CXCR7-agonist VUF11207 reduces phosphatidylserine exposure and the percentage of procoagulant platelets upon activation, without affecting the basal levels. Consequently, the presence of CXCR7-agonist counteracts activated platelet-driven procoagulant response, e.g., thrombin generation, or ADP-induced clot formation in thromboelastographic assays performed with human platelet-rich plasma and blood respectively. However, the CXCR7-agonist does not interfere with the basal hemostatic response or with plasma coagulation. Therefore, administration of CXCR7-agonist does not increase bleeding time in mice or affect coagulation profile in murine platelet poor plasma (activated partial thromboplastin time-APTT, pro-thrombin time-PT) [76]. The possibility of sparing basal hemostatic response but counteracting pathological thrombosis is seemingly an advantageous feature in such a novel anti-thrombotic strategy under consideration. 

#### 2.4.3. VUF11207 Counteracts Thrombo-Inflammatory Platelet Functions

Platelets are versatile cells which not only mediate hemostasis but also thrombo-inflammatory adversities that can exaggerate vascular and microvascular thrombosis in the veins [77], arteries [78], and pulmonary micro-capillaries [79]. Thrombo-inflammatory mediators released from activated platelets may influence the severity of organ damage during the acute inflammatory phase post-infarct, and also subsequent resolution of the inflammatory process, facilitating gradual regeneration, remodeling and functional recovery [80,81]. Moreover, platelet-derived soluble mediators (e.g., IL-1β,sCD40L) add to the circulatory levels from other cellular sources during acute inflammation [82,83] or chronic atheroprogression [84]. Therefore, thrombo-inflammatory attributes of platelets are being extensively investigated to develop novel therapeutic interventions for cardiovascular disease [2,81,85], and immunothrombotic complications instigated by FcγRIIA-mediated [86] activation of platelets. Platelet-leukocyte associations [87] play a significant role in aggravating thrombotic complications [82,85]. Increased formation of platelet-monocyte aggregates are observed in the circulation of ACS patients [88], attributed to elevated levels of pro-inflammatory sP-selectin, IL-6, which confers an increased risk of ACS. Similarly, platelet–neutrophil interactions in heparin induced thrombocytopenia-(HIT) [77] enhance thrombotic disposition. Considering the need for innovative anti-platelet approaches [81,84,85], the inefficacy of aspirin [89] in reducing immunothrombosis-associated mortality, further research is required in this direction. 

In our ongoing investigation, we observed that CXCR7-agonist inhibits formation of platelet-leukocyte aggregates in the effluent blood from flow chamber assay with human and murine blood ex vivo (Figure 2), closely resembling physiological conditions whereby activated platelets in circulation engage in thrombo-inflammatory associations with leukocytes. These results have been validated by assessing the impact of VUF11207 administration on circulatory platelet-leukocyte interactions 24 h post-MI/RI and following carotid artery injury in mice. VUF11207-administered mice show a significantly reduced platelet-leukocyte aggregate formation in peripheral circulation post-MI and arterial injury [76]. Increased plasma levels of IL-6 supports platelet–monocyte interactions in ACS patients [88]. CXCR7-agonist-administered mice also show significantly lower plasma levels of several inflammatory mediators (IL1α, IL1β, TNFα, IFNγ, IL-6, MCP-1) post-MI, which suggests its therapeutic benefits. In vitro, pre-treatment with VUF11207 reduces thrombo-inflammatory release from CRP-activated human and thrombin-activated murine platelets [76]. The therapeutic potential of CXCR7-agonist in a profound thrombo-inflammatory disease setting like HIT is also commendable. HIT is a drug-induced adversity that may lead to venous/arterial thrombosis. Upon heparin administration in emergency, susceptible individuals may develop anti-PF4-heparin antibodies that activate platelets through FcγRIIA, necessitating the replacement of heparin. CXCR7-agonist not only counteracts HIT-IgG-induced ex vivo activation of platelets (CD62P surface exposure, α_IIb_β_3_-integrin activation, thrombus formation), and interferes with platelet response in the heparin-induced platelet aggregation (HIPA) test, but also retards the release of thrombo-inflammatory IL-1β, IFN-γ, sCD40L, TNF-α, sP-selectin, the neutrophil chemoattractant IL-8, and thrombogenic tissue factor from activated human platelets. Neutrophils substantially aggravate HIT-associated thrombotic complications [77]. Neutrophils express ACKR3/CXCR7 [90,91], the antagonism of which regulates pulmonary injury associated with acute inflammation [92]. However, we observed that the CXCR7-agonist reduces HIT-IgG-induced human neutrophil activation, particularly the activation of CD11b in the Mac-1 complex. Thus, combined with its effect on platelet CD62P, CXCR7-agonist significantly reduces the formation of platelet-neutrophil aggregates triggered by HIT-IgG/sera [76]. These anti-thrombo-inflammatory attributes taken together suggests that therapeutics targeting ACKR3/CXCR7 may be effective in coping with HIT-associated thrombo-inflammation, also in acute inflammatory pathologies that trigger platelet FcγRIIA, as recently exemplified by vaccine-induced immune thrombotic thrombocytopenia-(VITT) [93] that closely resembles HIT, or antibodies generated in response to SARS-CoV2 infection [82,86]. 

#### 2.4.4. Unexpected Influence of ACKR3/CXCR7 on the Platelet Lipidome

**Figure 3:**
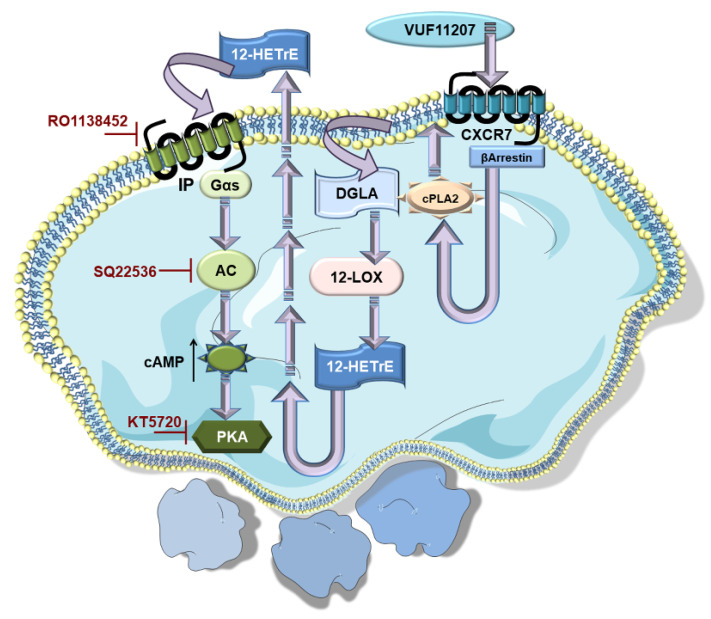
Non-canonical ACKR3/CXCR7 co-ordinates with canonical Gαs-coupled IP-receptor to impose platelet inhibition. ACKR3/CXCR7-ligation by a pharmacological agonist (VUF11207) modulates the platelet lipidome, leading to an increased generation of anti-platelet lipid DGLA.12-LOX metabolizes DGLA into 12-HETrE, its anti-platelet oxylipin derivative. 12-HETrE released in the microenvironment engages the Gαs-coupled IP receptor on platelets. This triggers the platelet inhibitory AC-cAMP-PKA signaling cascade, as adenylyl cyclase (AC) is activated to elevate cAMP levels and triggers the downstream cyclic nucleotide dependent protein kinase A (PKA). Therefore, platelet inhibitory effects of CXCR7-agonist are reduced in the presence of pharmacological interventions in the form of IP-receptor antagonist (RO1138452), AC (SQ22536), and PKA (KT5720) inhibitors.

Mechanistic insights into the anti-thrombotic actions of ACKR3/CXCR7 reveals an unexpected influence on both basal and thrombin-activated human platelet lipidome. ACKR3/CXCR7-ligation preferentially limits the metabolism and release of cyclooxygenase (COX-1) (thromboxane A2-TXA_2_) and lipoxygenase (12-LOX) (12-hydroxyeicosatetraenoic acid, 12-HETE) derived pro-thrombotic oxylipins, and phospholipase derived atherogenic lipid mediators like lysophosphatidylcholine (LPCs), while favoring the generation of anti-platelet lipids like dihomo-γ-linolenic acid (DGLA) derived 12-hydroxyeicosatrienoic acid (12-HETrE) [94], linoleic acid derived 13-Hydroxyoctadecadienoic acid (13-HODE), eisocapentaenoic acid (EPA) derived 12-Hydroxyeicosapentaenoic acid (12-HEPE) [95]. Platelets from CAD patients show increased levels of oxidized phospholipids, ceramides, sphingomyelins, triglycerides, diacylglycerols (DG), LPCs, and lysophosphatidylinositol (LPIs) [63], notwithstanding anti-platelet or statin therapy [76,96]. CXCR7-agonist counters the generation of arachidonic acid (AA), *12*-hydroxyheptadecatrenoic acid (12-HHT), TxA_2_, 12-HETE, LPIs, and DGs from thrombin activated platelets of healthy subjects and CAD patients ex vivo. This suggests the therapeutic potential of ACKR3/CXCR7 in regulating the generation of intracellular signaling intermediates (LPIs, DGs) that modulate the course and extent of platelet activation or lipid agonists (AA, TxA_2_) that perpetuate platelet aggregation [76]. As a consequence, CXCR7-agonist reduces intraplatelet calcium mobilization, phosphorylation of effector kinases in the platelet activatory signaling cascade, otherwise triggered by platelet stimulation. Such a unique influence of ACKR3/CXCR7 may check atherothrombosis by regulating the generation of pro-thrombotic and atherogenic lipids carried by platelets and platelet-derived microvesicles to the site of vascular injury or inflammation [97]. Future investigations will delineate the mechanistic basis behind this preference for 12-LOX, COX-1 and Cytp450 mediated metabolism of anti-platelet lipids, while limiting the generation of pro-thrombotic metabolites. 12-HETrE is a known anti-platelet mediator, which may be particularly effective in balancing the actions of 12-LOX-derived pro-thrombotic metabolite 12-HETE during platelet activation mediated through FcγRIIA [94,98,99]. Attributed to 12-HETrE, atypical ACKR3/CXCR7-ligation coordinates with the Gαs-coupled IP receptor to elevate platelet inhibitory cAMP levels; an effect contrary to the pro-thrombotic actions of Gαi-coupled CXCR4 (Figure 1) [59,63,64]. Engagement of CXCR4 by CXCL12/SDF-1α distinctively reduces platelet inhibitory cAMP levels to support platelet activation [59]. On the contrary, the anti-platelet effects of CXCR7-agonist are counteracted by IP-receptor antagonist and pharmacological inhibitors of the AC-cAMP-PKA pathway in both healthy donors and CAD patients ex vivo (Figure 3) [76]. This functional dichotomy between ACKR3/CXCR7 and CXCR4 (Figure 1) poses a unique physiological scenario whereby a balance between pro- and anti-thrombotic responses can be fine-tuned in CAD patients, in whom platelet surface expression of both CXCR4 and ACKR3/CXCR7 are enhanced. 

## 3. Therapeutic Implication of ACKR3/CXCR7 in Platelet-Associated Cardiovascular Disease

**Figure 4:**
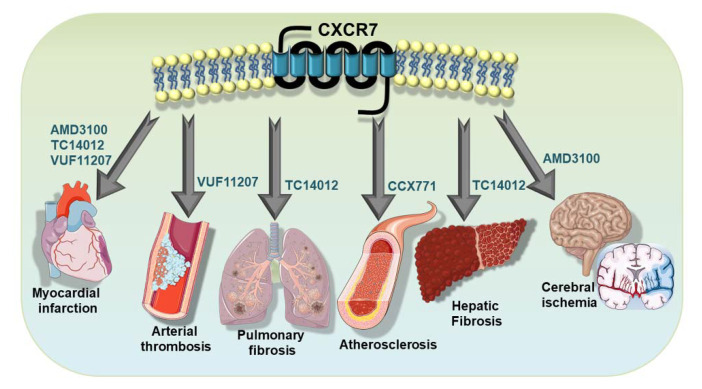
Therapeutic implementation of ACKR3/CXCR7 in platelet-associated pathologies. Therapeutic potential of ACKR3/CXCR7 has been demonstrated using pharmacological agonists in disease models of atheroprogression, arterial thrombosis, myocardial infarction, cerebral stroke, in impeding pulmonary fibrosis and in promoting hepatic regeneration.

Platelet-driven functional processes occupy an important niche in cardiovascular pathophysiology [4,5,7,80,81,100,101,102]. In recent years, the functional involvement of ACKR3/CXCR7 in cardiovascular disease has been delineated using a lineage-specific receptor deficient mouse lines [26,27,103,104,105] (Table 1). Therapeutic implications of targeting ACKR3/CXCR7 [46,106,107,108] using pharmacological agonists [107,109,110] (Table 2) have been verified in murine models of atheroprogression [103,111,112], which is intricately associated with inflammatory platelet functions. Several CXCR7-agonists have also been tested in arterial thrombosis [76]; in cerebral stroke [29,30,32,113,114,115], myocardial infarction/ischemic injury (MI/IR) and heart failure [26,27], diseases that are affected by thrombotic complications (Figure 4); and also in pulmonary [104] and hepatic fibrosis [105]. To this effect, various pharmacological CXCR7-agonists have been employed, e.g., cyclic-peptide-(TC14012) [104,105,109], allosteric agonist-(AMD3100) [27,110] ACKR3/CXCR7 specific CCX771 [103], and VUF11207 [76], demonstrating the therapeutic benefits of ACKR3/CXCR7 in functional recovery following myocardial infarction [27,28,76] in impeding pulmonary fibrosis [104], in promoting hepatic regeneration [105] and in retarding atheroprogression [103] (Table 2 and Figure 4). Although most of these investigations did not address the direct impact of pharmacological CXCR7 agonists on platelet response as we have done recently [76], they certainly verified the regenerative implication of ACKR3/CXCR7 in the target organs or tissues that are infiltrated by activated platelets and that are influenced by platelet response. 

### 3.1. Therapeutic Potential of ACKR3/CXCR7 against Atheroprogression 

ACKR3/CXCR7 is richly expressed at the macrophage-enriched aortic atheroma of atherosclerosis-prone *apoe*^−/−^ mice [111], and its expression is induced during human monocyte to macrophage differentiation [111] in vitro. Interestingly, atorvastatin exerting a pleiotropic effect independent of its cholesterol-lowering activity downregulates the expression of ACKR3/CXCR7 transcript in human THP-1 macrophages in vitro [112]. ACKR3/CXCR7 expression is observed in murine endothelial cells, which is significantly induced in the injured arteries and co-localizes with von Willebrand factor in neointimal endothelial cells. Similarly, CXCR7 expression is also observed in lesional endothelial cells of human aortic specimens. However, hyperlipidemic *apoe*^−/−^ mice ubiquitously and conditionally deficient in *Cxcr7* show increased numbers of monocytes in circulation, distinctive neointimal hyperplasia and macrophage accumulation at carotid lesions. Administration of a pharmacological CXCR7-agonist from ChemoCentryx, CCX771, decreases neointimal area, monocytosis, accumulation of Mac2^+^ macrophages at lesions, all of which contribute to ameliorate atheroprogression [103]. Apparently paradoxical, these findings heighten cell/tissue specific functional response under the regulatory influence of ACKR3/CXCR7. Administration of CCX771 for 4 weeks to hyperlipidemic mice reduces plasma levels of cholesterol by promoting the uptake of very low-density lipoproteins-(VLDL) but not LDL or high-density lipoprotein-(HDL) in the ACKR3/CXCR7 expressing white adipose tissue. CCX771 also increases lipoprotein lipase activity in the same by decreasing expression of the negative regulator of lipase activity *Angpt14* [103]. Like the pharmacological agonist CCX771, CXCL12/SDF-1α increases LDL and oxLDL uptake in platelets through active participation of both CXCR4 and ACKR3/CXCR7, since this process is counteracted by antibodies blocking either receptor. Treatment of human platelets with oxLDL in vitro drives platelet activation, degranulation and surface exposure of CXCL12/SDF-1α, an internalization of CXCR4, but surface translocation of ACKR3/CXCR7. Therefore, intraplatelet oxLDL level, which is significantly enhanced in ACS patients, correlates positively with platelet ACKR3/CXCR7 surface expression but inversely with that of CXCR4 [63]. Although these clinical and experimental evidences are of great interest, the molecular mechanism driving ACKR3/CXCR7 influenced lipid uptake and lipase activity in tissue (e.g., adipose) and cellular systems (e.g., platelets) remains to be ascertained. Substantiating the observation from Christian Weber’s group [103], subsequent investigators have also shown that both human and murine injured arteries show enhanced expression of ACKR3/CXCR7 among lesional endothelial cells, and endothelial cells of neointima, respectively [27]. Moreover, genetic ablation of ACKR3/CXCR7 from the endothelial lineage in *CXCR7*^f/f^*Cdh5-CreERT2*^+^ conditional knockout mice substantiates neointimal development following vessel injury, retards re-endothelialization and promotes atheroprogression [27]. 

Platelet-lipid or lipoprotein interactions may exert a significant impact in seeding atheroprogression [63,65,119]. Recently, we have observed a regulatory influence of ACKR3/CXCR7 in modulating the platelet lipidome. The presence of pharmacological CXCR7-agonist VUF11207 limited the generation of phospholipase C-derived LPCs [76] with distinct atherogenic properties in human platelets. LPCs are a major constituent of activated platelet-derived microvesicles. They are richly deposited at atherosclerotic plaques, and are demonstrated as a surrogate marker for plaque instability in hyperlipidemic (*apoe*^−/−^) mice [97]. Enrichment of LPCs is also observed in human endarterectomy specimens from patients with symptomatic carotid artery stenosis. By acting through the receptor G2AR, LPCs can induce platelet activation (CD62P surface expression α_IIb_β_3_-integrin activation), and prompt formation of platelet-monocyte aggregates, thus bearing the potential to drive atherothrombotic complications. A limitation on thrombin-induced generation of LPCs in human platelets, as offered by CXCR7-agonist VUF11207, may be considered advantageous over aspirin, which inhibits COX-1 downstream of phospholipases so that atherogenic LPCs can be generated and remain elevated in platelets from CAD patients, despite anti-platelet and statin therapy [63,76,96].

### 3.2. Therapeutic Efficacy of ACKR3/CXCR7 in Limiting Fibrosis and Promoting Tissue Regeneration

Platelets are involved in both reparative and fibrotic processes that follow tissue injury, having an array of soluble mediators ready to be unleashed from their granules. Substantial evidence from previous investigations point towards the regenerative inclination of ACKR3/CXCR7, while there is a more pro-fibrotic effect executed through CXCR4. Although a direct involvement and relative contribution of platelet CXCR4 and ACKR3/CXCR7 to such derogatory or reparative consequences remains to be seen, we can gain valuable insights from the available experimental and clinical evidences that have been accumulated over the years, as discussed in the context of myocardial, pulmonary and hepatic regeneration. 

#### 3.2.1. Myocardial Regeneration and Functional Recovery

GPCRs have a pivotal contribution in cardiac development and functioning, in both health and disease [120]. Therapeutics targeting β-arrestin-biased signaling through β-adrenergic (e.g., carvedilol) [121] and angiotensin II receptors (e.g., TRV120067) [122], are cardioprotective agents applied in clinical practice or being validated in preclinical studies. The CXCL12/SDF-1-CXCR4-CXCR7 axis has long since been recognized as a significant modulator of cardiac development, functioning, and repair. Both CXCR4 [123,124,125] and ACKR3/CXCR7 [126,127,128,129,130] are expressed by cardiomyocytes and exert complementary but differential influence on distinct aspects of cardiac development [131]. *Cxcr7* is the most abundantly expressed seven pass transmembrane receptor gene in the murine heart and functions as a β-arrestin-biased receptor [26]. *Cxcr7* mRNA levels are three-fold higher than those of the type 1A angiotensin II receptor (Agtr1a) and tenfold higher than those of the β1-adrenergic receptor (Adrb1). Single-cell RNA-sequencing for 23 chemokine receptor transcripts in various cell types of the murine heart shows that ACKR3/CXCR7 is abundantly expressed in cardiomyocytes, and cardiac fibroblasts [26]. ACKR3/CXCR7 plays a critical role in cardiac development as its ubiquitous deletion in *Cxcr7*^−/−^ mice causes postnatal lethality due to ventricular septal defects and malformation of the semilunar heart valve on account of diminished expression of factors essential for valve and vessel formation, growth and survival of endothelial cells [126]. Confirming this report, another group showed ACKR3/CXCR7 expression in murine cardiomyocytes, vascular endothelial cells of the lung and heart, the cerebral cortex and osteocytes, employing a LacZ reporter knock-in system. They also reported 70% postnatal lethality in *Cxcr7*^−/−^ mice within a week after birth. *Cxcr7*^−/−^ mice exhibit enlarged hearts, myocardial degeneration, postnatal fibrosis and hyperplasia of embryonic origin [130]. More recently smooth muscle specific ablation of *cxcr7* in SM22α-Cre^+^CXCL12^f/f^ mice has been reported to cause 50% perinatal mortality. Postnatally, these mice develop severe fibrotic hypertrophy, show impaired cardiac function, and also thin and dilated coronary arteries associated with diminished ACKR3/CXCR7 expression in the endothelial cells. However, pharmacological targeting of ACKR3/CXCR7 by administration of CXCR7-agonist TC14012 retards cardiac hypertrophy in these mice, suggesting its therapeutic efficacy in counteracting adverse remodeling. TC14012-treated SM22α-Cre^+^CXCL12^f/f^ mice also show improvements in LVEF [132].

CXCR4 expression is increased in the infarct region following MI as established from PET/CT imaging studies in murine models and humans [118,133]. The cardioprotective effects of CXCR4 antagonist but allosteric CXCR7-agonist AMD3100 is well acknowledged in counteracting the actions of CXCL12/SDF-1α-CXCR4 axis during the acute phase of myocardial remodeling. AMD3100 reduces infarct size, non-infarcted left ventricular hypertrophy, improves systolic function [116,134], and decreases scar expansion. Since CXCR4 regulates retention of progenitor cells in the bone marrow, CXCR4-antagonism by AMD3100 augments mobilization of bone marrow-derived EPCs into circulation, their cardiac engraftment and EPC facilitated neovascularization. This reduces fibrosis and preserves myocardial function following MI [117,135,136]. In recent years, the therapeutic benefits of CXCR7-specific agonist TC14012 [27,28] has also been demonstrated to improve myocardial regeneration and functional recovery following MI [137,138]. 

Expression of ACKR3/CXCR7 is significantly increased at the infarct border zone than in the remote areas, along with concomitant activation-induced phosphorylation of Erk, a signature signaling intermediate in the CXCL12/SDF-1α-CXCR7 pathway [26]. Single-cardiomyocyte RNA-seq results from human heart biopsies reveal an increased CXCR7 expression in the cardiomyocytes of heart failure patients. This phenotype is reverted back to normal upon left ventricular assist device (LVAD) implantation in both ischemic and non-ischemic cardiomyopathies. These observations confirm the clinical relevance of ACKR3/CXCR7 in myocardial pathophysiology and the possibility of pre-clinical validation in murine models. This investigation further showed that cardiomyocyte-specific deletion of *cxcr7* in αMHC-Cre^+/−^ CXCR7^flox/flox^ conditional knock out mice results in a more prominent left ventricular enlargement and systolic dysfunction following MI [26]. Conditional deletion of *cxcr7* from the endothelial lineage in CXCR7^f/f^Cdh5-CreERT2^+^ mice also increases infarct size, causes functional impairment, reduces vascular density in the infarcted region and promotes fibrotic remodeling following MI [27]. The investigators have postulated that in the absence of ACKR3/CXCR7, circulatory CXCL12/SDF1α, although it has its regenerative attributes, could affect post-MI cardiac remodeling [27] by engaging CXCR4, which is known to mediate fibrotic consequences. Targeted (adenoviral) delivery of *cxcr7* to the left ventricle and administration of CXCR7 agonist TC14012 [27] offers cardio-protective benefits. This therapeutic effect has been confirmed by other investigators using TC14012 [28], and recently by us using VUF11207 [76]. C57BL/6 J mice treated with TC14012 (i.p.) following MI enhances the angiogenic response in the ischemic heart tissue ascertained by increased expression of vWF, VEGFR2, p-SRC, p-PLC-γ and the p-P38/t-P38 ratio. TC14012 counteracts the deterioration in cardiac function (e.g., ejection fraction and fractional shortening), and reduces the infarct size after AMI [28]. Similarly, VUF11027 administered (i.v.) mice show a reduced infarct size and less deteriorated LVEF 24 h post-MI, concomitantly with reduced platelet-driven thrombotic response and thrombo-inflammatory platelet-leukocyte interaction in circulation [76]. However, systemic administration of VUF11207 may have a significant impact exerted on both the myocardium and peripheral blood cells, including platelets, which express ACKR3/CXCR7 and variously contribute to post-infarct myocardial recovery and remodeling [139,140,141,142]. Anti-platelet therapies targeting platelet COX-1 (aspirin), P_2_Y_12_ (clopidogrel, ticagrelor, prasugrel, cangrelor), GPIIbIIIa (abciximab, tirofiban, eptifibatide), GPVI (revacept),p-selectin (fucoidan, inclacumab) can exhibit pleotropic cardioprotective effects [5]. Novel theranostics in preclinical validation that target activated platelets to enrich delivery of cardioprotective drugs (Targ-CD39) [34,143] or progenitor cells (Tandem-scFv_GPIIb/IIIa-Sca-1_) [144] effectively combine diagnostic tools [145,146] and therapeutic prospects [33,34,144] for myocardial regeneration and functional recovery in murine models. However, these innovative and clinical therapeutics exclusively target platelet receptors, while ACKR3/CXCR7 is broadly expressed in the ischiatic cardiac microenvironment, including infiltrating cells. Administration of VUF11207 pre-MI, counteracts subsequent platelet activation (p-selectin exposure, GPIIbIIIa activation) that influences platelet infiltration into the affected myocardium, thrombotic propensity and reduces platelet-leukocyte aggregates in circulation 24 h post-MI, which may otherwise aggravate acute inflammatory processes following reperfusion. However, currently observed myocardial benefits of VUF11207 cannot be directly or entirely accredited to its anti-platelet action. The anti-thrombotic effects of VUF11207 possibly adds to its therapeutic value exerted directly on the myocardium post-MI [76], as previously demonstrated in case of TC14012 [27,28] since cardiomyocyte ACKR3/CXCR7 expression is increased. Further investigations are necessary to delineate the significance and involvement of platelet ACKR3/CXCR7 in modulating their interactions with other cellular components of the ischemic myocardium. To what extent platelet-ACKR3/CXCR7 may complement, override or impede the regenerative impact of cardiomyocyte-ACKR3/CXCR7 remains to be ascertained. Nevertheless, current investigations reinforce the therapeutic potential of ACKR3/CXCR7 in restoring the functionality of the affected myocardium, particularly since enhanced ACKR3/CXCR7 expression on both cardiomyocytes and circulating platelets offers a readily available therapeutic target. 

#### 3.2.2. Pulmonary Fibrosis 

In the hematopoietic-vascular niche of the lungs, ACKR3/CXCR7 provides an in-built regenerative mechanism to prevent fibrosis following pulmonary injury. ACKR3/CXCR7 is expressed in the pulmonary capillary endothelial cells (PCECs), actively prevents epithelial damage and retards fibrosis following a single phase intratracheal injection of bleomycin or hydrochloric acid. However, with repeated injury, ACKR3/CXCR7 expression is suppressed and the pro-fibrotic mechanism takes over, involving the recruitment of perivascular macrophages. This ensues a persistent upregulation of Notch signaling in the PCECs and perivascular fibroblasts to promote lung fibrosis. Incidentally, ACKR3/CXCR7 expression is also reduced in patients with interstitial pulmonary fibrosis [104]. Intratracheal administration of the CXCR7-agonist TC14012 following injury prevents alveolar epithelial damage and reduces pulmonary fibrosis. TC14012 counteracts pro-fibrotic response like β-catenin-dependent induction of Notch ligand Jag1 expression in PCECs, and Hes1 in pulmonary fibroblasts, and also amends increased levels of α-smooth muscle actin (SMA) and collagen I synthesis in the injured lung [104]. 

The thrombo-inflammatory impact of platelets in respiratory diseases is well investigated and characterized in acute infectious pulmonary diseases inflicted by *Streptococcus pneumoniae* [147,148], *Pseudomonas aeruginosa* [149], influenza A virus H1N1 (A/H1N1) [150,151], severe acute respiratory syndrome coronavirus 2 (SARS-CoV-2) [85,86,87]. Platelets also effectively contribute to chronic respiratory diseases like asthma [152,153], chronic obstructive pulmonary disease (COPD) [154] and aspirin-exacerbated respiratory disease (AERD) [155]. Platelet-derived inflammatory mediators, platelet interaction with inflammatory cells of the innate (monocyte, neutrophils) or the adaptive immune wing play a decisive role in disease progression and severity [156]. Some of these pathologies involve platelet activation through FcγRIIa as an effector mechanism in instigating a thrombo-inflammatory response [85,86], while platelet derived TxA_2_ may induce bronchoconstriction [157] and cysteinyl leukotrienes produced during transcellular lipid metabolism involving platelets and leukocytes [158] can exaggerate the inflammatory processes. The link between inflammation and thrombosis is infallible in cardiovascular disease. The impact of anti-platelet therapies on circulatory inflammatory mediators like hsCRP, IL-6, and TNF-α has been deduced in clinical studies [80]. Anti-platelet therapeutics like cangrelor are effective against pulmonary fibrosis in a murine model [159]. Given the already recognized influence of CXCR7-agonist in ameliorating pulmonary fibrosis [104], its recently discovered efficacy in governing thrombo-inflammatory platelet-leukocyte interactions, and its regulation on the generation of thrombo-inflammatory lipids (e.g., TxA_2_, 12-HETE) [76], the anti-platelet significance of ACKR3/CXCR7 deserves a closer look in chronic and acute pulmonary inflammation.

#### 3.2.3. Hepatic Regeneration 

The commendable regenerative potential of the liver [160] may be surmounted by a fibrotic response following chronic and acute hepatic inflammation or injury that can eventually lead to hepatic cirrhosis and failure [161,162,163]. The hepatic vascular niche includes liver sinusoidal endothelial cells (LSECs), which are primarily responsible for regeneration [160] through the release of angiocrine factors, whereas the non-parenchymal hepatic stellate cells (HSC) mediate extracellular matrix deposition, leading to fibrosis [163]. As observed for platelets [76], a striking functional dichotomy of CXCR4-CXCR7 has been demonstrated in balancing the regenerative as opposed to the fibrotic phenotype of LSECs following acute and chronic liver injury [105]. The transition from a regenerative to a pro-fibrotic hepato-vascular niche involves differential expression of ACKR3/CXCR7 and CXCR4 in the LSECs. Acute hepatic injury inflicted by carbon tetrachloride (CCl_4_) and acetaminophen causes upregulation in the expression and functional engagement of CXCR4, ACKR3/CXCR7 in LSEC that triggers generation of regenerative angiocrine factors. ACKR3/CXCR7 expression is primarily upregulated in VE-cadherin^+^ LSECs, whereas CXCR4 is stably and broadly expressed by different cell types. Both physiological (CXCL12/SDF-1α) and pharmacological (TC14102) CXCR7-agonists induce expression of the transcription factor-Id1 in cultured LSECs. Moreover, inducible endothelial deletion of *cxcr7* in LSECs of Cxcr7^iΔEC/iΔEC^ mice impairs liver regeneration by decreasing Id1-induced production of angiocrine factors. However, chronic insult with repetitive hepatotoxin (carbon tetrachloride) injection and bile duct ligation increases CXCR4 expression, as the pro-fibrotic FGFR1/CXCR4 angiocrine pathway counters the regenerative potential of ACKR3/CXCR7. Levels of pro-fibrotic α-SMA and collagen are particularly elevated in the absence of *cxcr7* from the endothelial lineage in Cxcr7^iΔEC/iΔEC^ mice. Administration of CXCR7-agonist TC14102 retards these pro-fibrotic changes, demonstrating its therapeutic potential to ameliorate hepatic fibrosis and to promote regenerative mechanisms [105]. Another investigation employing a partial hepatectomy model in rats demonstrated that expression of CXCL12/SDF-1α is significantly upregulated in LSECs, which mediates proliferation, mobilization, and engraftment of ACKR3/CXCR7^+^ progenitors of LSECs to facilitate hepatic regeneration [164].

Platelets have a very close association with the pathophysiological functioning of the liver [165,166], since both platelet biogenesis and clearance of senescent platelets are influenced or operated by cellular systems in the liver [167]. Liver parenchymal and sinusoidal endothelial cells synthesize and release the hormone thrombopoietin (TPO), which boosts megakaryopoiesis to generate platelets. Therefore, hepatic malfunctioning in diverse liver-related pathologies is associated with a drop in circulatory platelet count. Platelets on the other hand contribute to the maintenance of physiological hemostasis and vascular integrity by acting as sentinels detecting liver infection, inflammation and injury. Platelets actively contribute to several liver pathologies [168], including non-alcoholic fatty liver diseases [169], liver inflammation and hepatitis [170]. Platelet-derived functionally opposing fibrotic (serotonin-5-HT, sphingosine-1-phosphate, platelet factor 4-CXCL4) and anti-fibrotic (ADP, ATP, hepatocyte growth factor) granular releasate can regulate the propensity of hepatic stellate cells to synthesize extracellular matrix components like collagen I and II, or retard the process respectively, besides promoting fibrinolysis by enhancing matrix-metalloproteinase (MMP) activity [165]. Platelets are recruited to LSECs following chemical injury (CCl4) or hepatectomy. Platelet-derived CXCL12/SDF-1α stimulates ACKR3/CXCR7^+^ liver LSECs and contributes to hepatic regeneration by inducing the production of pro-regenerative angiocrine Wnt2 and HGF. This process is impaired in thrombopoietin-deficient *Thpo*^−/−^ mice with suppressed circulatory platelet counts and following induced deletion of *Cxcr7* in LSEC of *Cxcr7*^iΔ/iΔ^ mice [171]. The contribution of platelet ACKR3/CXCR7 in influencing their interaction with the hepatic microenvironment is warranted. ACKR3/CXCR7 being a prosurvival receptor for platelets, research in this direction might reveal valuable insights into the hepatic trigger for platelet biogenesis and clearance of aged platelets in the hepatic vascular niche, besides modulating their active participation in hepatic immune surveillance and regeneration. 

## 4. Discussion

### ACKR3/CXCR7 as an Anti-Thrombotic Drug Target: Considering the Pros and Cons

**Figure 5:**
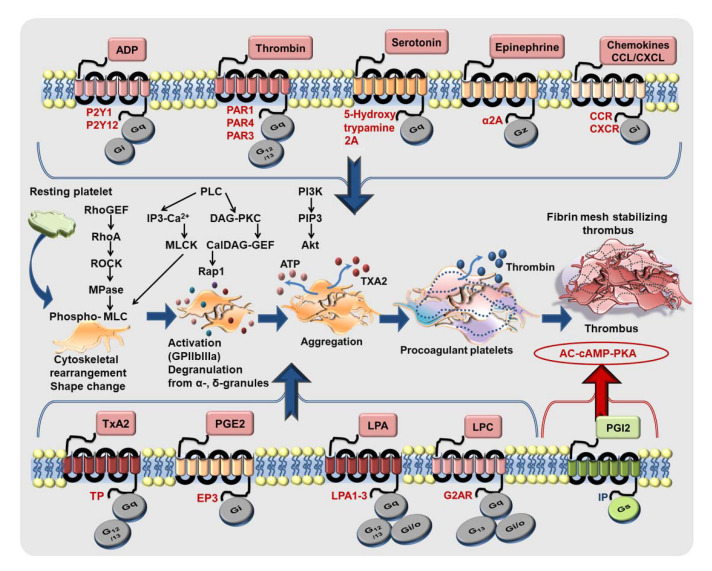
*GPCRs driving platelet response to activating* stimuli. Platelet-activating pathophysiological stimuli (nucleotides, protease, peptides, lipid agonists, amines, chemokines) engage several GPCRs coupled with diverse G-proteins (G_q_, G_12/13_, G_i,_ G_z_) to orchestrate platelet shape change, activation, degranulation, aggregation, procoagulant function leading to thrombus formation. Lipid agonists thromboxane A_2_ (TxA_2_) and prostaglandin E_2_ (PGE_2_), lysophatidylcholine (LPC) and lysophosphatidic acid (LPA) also propagate platelet activation through GPCRs. Platelet activation downstream of different GPCRs is both extensive and intricate. However, they engage some common mediators like Rho-guanine nucleotide exchange factor (RhoGEF), calcium and diacyl glycerol-regulated guanine nucleotide exchange factor (CalDAG-GEF), myosin phosphatase (MPase), and signature signaling events involving phospholipase C (PLC) activation, generation of lipid signaling intermediates inositol 3 phosphate (IP3), diacylglycerol (DAG), phosphatidylinositol 3,4,5, trisphosphate (PIP3), intraplatelet calcium mobilization, myosin light chain (MLC) phosphorylation, activation of protein kinases, e.g., Rho-associated kinase (ROCK), myosin light chain kinase (MLCK), protein kinase C (PKC), phosphatidylinositol 3 kinase (PI3K), Akt. Platelet inhibitory prosglandin I_2_ (PGI_2_) also exerts its action through G_s_ coupled IP-receptor and triggers the platelet inhibitory signaling cascade involving adenylyl cyclase (AC), cyclic adenosine monophosphate (cAMP) and protein kinase A (PKA).

Platelet activation is mediated through engagement of several GPCRs by physiological agonist like P2Y1 (G_q_), P2Y12 (G_i_) by ADP; PAR1 (G_q_, G_12/13_ in human platelets), PAR4 (G_q_, G_12/13_ in human and murine platelets) by thrombin, thrombin receptor activating peptide (TRAP), and PAR4-agonistic peptide; TxA_2_ receptor-TP (G_q_, G_12/13_); α2A-adrenergic receptor (G_z_) by epinephrine; EP3 receptor (G_i_) by prostaglandin E2; 5-hydroxytryptamine A2 receptor (G_q_) by serotonin; chemokine receptors (G_i_) engaged by CXCL12/SDF1-α, MDC/CCL22, TARC/CCL17, RANTES/CCL5, MCP-1/CCL2, fractalkine/CX3CL1, which act as synergistic activating cues [172,173]. To briefly recapitulate, CXCL12/SDF1-α acts as a pro-thrombotic agent through G_i_ coupled CXCR4 bringing forth several downstream events characteristics of the activatory signaling cascade in platelets including PLC activation, calcium mobilization, activation of PI3K, Akt, MLC-phosphorylation, CD62P surface expression, GPIIbIIIa activation, release of ADP, TxA_2_, and a concomitant decrease in platelet inhibitory cAMP levels to drive platelet aggregation and thrombotic response. ACKR3/CXCR7, on the other end, being a non-canonical GPCR, does not fit into this scheme. Moreover, lysophosphatidic acid receptors (LPA_1–3_), receptors (G_q_, G_i/o_, G_12/13_), [174] and LPC receptor G2AR (G_q_, G_i/o_, G_13_) [97] are also involved in platelet activation (Figure 5). Therefore, platelet GPCRs constitute prime drug targets for therapeutic intervention and have been reviewed extensively [172,173,175,176,177]. Anti-platelet therapies like aspirin and P_2_Y_12_-antagonists are cornerstones in the prevention and secondary management of platelet hyper-reactivity that frequently leads to recurrent thrombo-ischemic complications in ACS patients [178]. Better anti-thrombotic strategies are required to overcome the drawbacks of current therapeutics, which are manifested as increased bleeding complications upon prolonged use, or refractoriness to optimal therapeutic benefits [179,180].

Platelet surface expression of ACKR3/CXCR7 is low under physiological conditions [37,40], which can be dynamically altered in the presence of CXCL12/SDF1-α. However, levels of pro-inflammatory mediators like CXCL12/SDF1-α and MIF are also low under physiological conditions, and are therefore unlikely to alter ACKR3/CXCR7 surface availability or trigger ACKR3/CXCR7-mediated regulatory effects. However, plasma levels of CXCL12/SDF1-α [12,38] and MIF [45] are elevated in CAD patients, which, combined with increased surface availability of both CXCR4 and ACKR3/CXCR7 on platelets [23,25], may decide on the balance between pro- and anti-thrombotic functions. ACKR3/CXCR7 does not play an active role in mediating thrombotic-hemostatic platelet response like other GPCRs (Figure 5), e.g., PARs, P_2_Y_12_, P_2_Y_1_, which are engaged by platelet-activating stimuli thrombin and ADP, respectively. To date, there is no experimental evidence suggesting a potential crosstalk between ACKR3/CXCR7 and receptors that either execute a thrombotic response, i.e., the glycoproteins-GPI, GPV, GPVI, GPIX, the PARs, purinergic receptors, or those that inhibit platelet function, i.e., immunoreceptor tyrosine-based inhibitory motif (ITIM) bearing receptors. In vivo administration of CXCR7-agonist (VUF11207) does not affect the surface availability of GPIb, GPV, GPVI, GPIX, α_v_- β_3_-integrin on murine platelets [76]. Currently, we do not have any reason to speculate that ACKR3/CXCR7 may directly sequester or influence ligand binding to any of these receptors. However, as a high affinity “decoy” receptor, ACKR3/CXCR7 may sequester and degrade CXCL12/SDF1-α [108,181,182,183,184,185,186], preventing its pro-thrombotic actions through CXCR4 [15,21,59,64]; but then again, levels of CXCL12/SDF1-α and platelet surface expression of ACKR3/CXCR7 are both low under physiological conditions. Taking these possibilities into consideration, it is evident that any regulatory influence mediated through ACKR3/CXCR7 on platelet surface can only be triggered in the presence of a receptor-specific inflammatory (CXCL11, CXCL12/SDF1-α, MIF) or pharmacological ligand, and that genetic deletion of ACKR3/CXCR7 might not affect basal platelet responsiveness or exhibit a striking platelet-centered phenotype in vivo. Nevertheless, unlike anti-platelet drugs in clinical practice, ACKR3/CXCR7 offers us the possibility of fine-tuning platelet response without interfering with the availability or functionality of receptors that sense vascular damage/alterations (GPIb, GPV, GPVI, GPIX) or those that mediate physiological hemostasis (e.g., integrins, PARs, P_2_Y_12_). 

The known physiological ligands of ACKR3/CXCR7 are either pro-inflammatory chemokines (CXCL11/ITAC, CXCL12/SDF-1α), cytokines (MIF) or adrenomedullin; therefore, they cannot be intended for therapeutic purposes. To date, a number of ACKR3/CXCR7 agonists have been invented including peptide agonists, cyclic peptides, small molecule agonists and even orally available natural agonists [187] that exhibit different degrees of specificity (Table 2) and an affinity for ACKR3/CXCR7 [106,107,108,188,189]. Use of these agonists for preclinical validation of ACKR3/CXCR7 as an anti-platelet drug target is definitely plausible. Some of these agonists are convincingly effective in ameliorating cardio/cerebrovascular complications [26,27,28,103] and vascular pathologies (Figure 4). However, currently available ACKR3/CXCR7 agonists or those in development will have to consider the following aspects and not just pharmacologic, pharmacokinetic and regulatory toxicological measures, before their translational implementation as an anti-platelet therapy in clinical practice.

- ACKR3/CXCR7 is ubiquitously expressed to various extent by circulating immune (monocytes, macrophages, [28] T-lymphocytes [190], B-lymphocytes [191], neutrophils [90,91,92]) and vascular cells (endothelium [27]) and also cells constituting the organs that these blood vessels perfuse (e.g., myocardium [26], brain [30,32,192,193]). Therefore, ACKR3/CXCR7 may elicit a cell- and organ-specific functional response [46], which cannot be restricted specifically to platelets. This necessitates a thorough functional characterization of anticipated cellular targets under the influence of a CXCR7-agonist, to prevent undesirable off-target deleterious effects other than the one intended as an anti-platelet agent.
- It is absolutely essential to consider that ACKR3/CXCR7 exhibits a ligand-specific functional response [194] even in platelets. Although CXCL11, CXCL12/SDF1-α and MIF execute an anti-apoptotic effect through ACKR3/CXCR7, only MIF demonstrates an anti-thrombotic influence [37,40], although this is without affecting other platelet functions like spreading, degranulation and aggregation [74,75]. CXCL12/SDF1-α evidently promotes a pro-thrombotic response but through CXCR4 [21,59,64]. Such discrepancies may arise due to ligand-specific conformational changes upon ACKR3/CXCR7 engagement [194]. A pharmacological CXCR7-agonist intended as an anti-platelet agent will have to be validated for several platelet functions, their interaction with circulatory and vascular cells, and those in the organs (e.g., myocardium) that are infiltrated by activated platelets [5,7,34], to fully apprehend its scope for therapeutic application in cardiovascular pathologies.
- Needless to say, that a detailed mechanistic insight into the anti-platelet mode of action is mandatory to convincingly attribute the observed anti-thrombotic benefits of existing CXCR7-agonists (Table 2) [106,107,109,110,189] or those in development, to be specifically mediated through ACKR3/CXCR7 and not as a pleiotropic effect.
- Over 90% of publications concerning ACKR3/CXCR7 enlisted in PubMed stem from research in the context of various cancers, where ACKR3/CXCR7 has been shown to play a decisive role in cancer progression, angiogenesis and even prognostic outcome [46,195,196,197]. Given the level of our current scientific understanding on ACKR3/CXCR7, it is perhaps neither advisable nor reasonable to continuously trigger this receptor by the prolonged use of an agonist. Therefore, it is essential to ascertain the therapeutic ‘time-window’ during which availability of ACKR3/CXCR7 is observed as a potential anti-platelet drug target in cardiovascular diseases, and to categorically define a dosage and treatment regimen. Our aim should be to utilize the therapeutic benefits of the ACKR3/CXCR7 agonist as an anti-thrombotic agent without inflicting neoplastic changes, and while doing so, to substantiate a functional recovery of the afflicted organs (e.g., myocardium) following thrombo-inflammatory and thrombo-ischemic damage, as effectively demonstrated for myocardial infarction [27,28,76]. The prospect of a ligand and cell-specific response, also biased signaling through ACKR3/CXCR7, can be exploited to fulfill this objective. However, fundamental research on the molecular mechanisms driving signaling events and functional response downstream of ACKR3/CXCR7 needs to be attended to in parallel with translational research initiatives to make productive progress in this direction.

## 5. Conclusions and Future Directions

Considering the versatile involvement of ACKR3/CXCR7 driving a miscellaneous cellular response in diverse pathologies and the multifaceted molecular pathways triggered [198], it is undoubtedly a challenging GPCR to work with. Some investigations have presented conflicting evidence on its expression or the lack of it in circulatory cells [199]. Its role has been defined merely as a “decoy receptor” for CXCL12/SDF1-α to a functional signaling receptor actively engaged in cellular growth, differentiation, survival, inflammation, and neoplastic transformation. Its involvement has been depicted in mediating various processes of tissue and organ regeneration [46,90,106,108,200]. The controversy about its restriction to atypical or non-canonical G-protein independent actions [181,201,202] and its potential to heterodimerize with and regulate canonical actions of CXCR4 [198], have further added to the challenges in delineating the molecular mechanisms instigated exclusively by ACKR3/CXCR7. There have been a little more than 900 articles retrieved from PubMed to date with the keyword “CXCR7”, since its discovery in 2005 [203], the bulk of which are investigations concerning cancer biology. Nevertheless, gathering evidence on the therapeutic implication of ACKR3/CXCR7 in atherosclerosis [103], myocardial infarction [27,28], pulmonary fibrosis [104], hepatic regeneration [105], its role in platelet pathophysiology and CAD [23,24,25,63] cannot be overlooked. Therefore, surmounting the challenges of investigating ACKR3/CXCR7 in experimental, preclinical and clinical studies possibly ensures an encouraging translational outcome.

## Figures and Tables

**Table 1:** Relevance of ACKR3/CXCR7 in vascular pathophysiology.

(Cardio) Vascular Pathophysiology	Animal Models Used	Mouse Line	Functional Evaluation	Ref.
**Atherosclerosis**	Wire injury of carotid arteries	*CAG-CreER^TM^Cxcr7^flox/flox^*, *CAG-CreER^TM^Cxcr7^flox/flox^Apoe*^−/−^ (ubiquitous *cxcr7* deletion)	Increased neointima formation, lesional macrophage accumulation after vascular injury.	[103]
			Increased serum cholesterol levels and hyperlipidemia-induced monocytosis.	
**Neointimal hyperplasia**	Endothelial denudation of the femoral artery by angioplasty wire inflicted injury	*CXCR7*^f/f^*Cdh5-CreERT2*^+^ conditional endothelial *cxcr7* deficient mice	Increased neointimal hyperplasia and neointima/media thickness ratio	[27]
**Angiogenesis**	Hind-limb ischemia (femoral artery ligation, laser Doppler imaging)	*CXCR7* ^f/f^ *Cdh5-CreERT2* ^+^	Reduced blood flow recovery, reduced vascular density in the ischemic gastrocnemius	[27]
**Pulmonary fibrosis**	Acute and chronic Intratracheal administration of bleomycin or hydrochloric acid	VE-cadherin–Cre^ERT2^*Cxcr7*^loxP/loxP^, *Cxcr7*^iΔEC/iΔEC^	Intra-tracheal instillation of TC14012 reduces pulmonary fibrosis (SMA, collagen I expression) and notch signaling in control mice	[104]
**Pulmonary inflammation**	Mice exposed to nebulized LPS; CXCR7 antagonist CCX771 (10 mg/kg body weight, s.c.)	C57BL/6 mice	Reduced transepithelial migration, release of neutrophil chemoattractant, neutrophil MPO activity and oxidative burst; decreased microvascular permeability in treated mice	[91]
**Hepatic fibrosis**	Single and repeated injections of CCl_4_ for acute and chronic injury; bile duct ligation induced cholestasis model	*VE-cad*-*Cre^ERT2^Cxcr7*^loxP/loxP^ Endothelial *cxcr7* knock out	Impaired hepatic regeneration due to diminished Id1-mediated generation of angiocrine factors; increased fibrotic response	[105]
**Myocardial infarction**	Permanent ligation of the left anterior descending (LAD) coronary artery	*CXCR7*^f/f^*Cdh5-CreERT2*^+^ inducible endothelial *cxcr7* knockout mice	Increased infarct size, reduced vascular density at the infarcted region, impaired cardiac function and remodeling post-MI, increased mortality	[27]
	Adenoviral delivery of *cxcr7* via left ventricle	C57BL/6 mice	Decreased infarct size, improved cardiac function	[27]
	LAD ligation	αMHC-Cre^+/−^ CXCR7^flox/flox^; cardiomyocyte-specific *Cxcr7* knockout mice	Normal heart phenotype but prominent left ventricular enlargement and systolic dysfunction post MI	[26]
	LAD ligation	Col1a2-CreERT2^+/−^ CXCR7^flox/flox^; fibroblast-specific knockout mice	No significant reduction in heart *Cxcr7* expression, weight, left ventricular volume, and systolic function under basal condition or after MI	[26]
	LAD ligation for 30 min, MI/IR; VUF11207 (i.v. pre-MI)	C57BL/6 mice	Reduced infarct size, less deteriorated LVEF 24 h post MI/IR	[76]

**Table 2:** Currently available CXCR7-agonists for pre-clinical validation in cardiovascular disease.

CXCR7 Agonist	Type	Tested Therapeutic Potential in	Ref.
VUF11207	Small molecule agonist	Platelet-inhibition; reduced arterial thrombosis	[76]
		Reduced thrombo-inflammatory response post MI/IR, arterial injury, that induced by HIT-IgG ex vivo	
		Reduced infarct size, less deteriorated LVEF post-MI	
ChemoCentryx CCX771	Small molecule agonist	Reduced atheroprogression following vascular injury in hyperlipidemic *Apoe*^−/−^ mice	[103]
		Reduced serum cholesterol and triglyceride levels in hyperlipidemic *Ldlr*^−/−^ mice	
TC14012	Cyclic peptide agonist	Myocardial regeneration, functional recovery post-MI	[27]
		Neovascularization and myocardial regeneration post-MI	[28]
		Reduced pulmonary fibrosis	[104]
		Improved hepatic regeneration, reduced fibrosis	[105]
AMD3100	Small molecule allosteric agonist	Myocardial recovery post-MI	[116,117,118]
		Reduced microglial activation, improved outcome following experimental ischemic stroke	[115]

## Data Availability

Not applicable.
